# Is arthroscopic repair superior to biceps tenotomy and tenodesis for type II SLAP lesions? A meta-analysis of RCTs and observational studies

**DOI:** 10.1186/s13018-019-1096-y

**Published:** 2019-02-13

**Authors:** Yi-Ming Ren, Yuan-Hui Duan, Yun-Bo Sun, Tao Yang, Wei-Yu Hou, Meng-Qiang Tian

**Affiliations:** Department of Joint and Sport Medicine, Tianjin Union Medical Center, Jieyuan Road 190, Hongqiao District, Tianjin, 300121 People’s Republic of China

**Keywords:** Rotator cuff, Tenotomy, Biceps tendon, Arthroscopy, SLAP lesion, Meta

## Abstract

**Objective:**

Labral repair and biceps tenotomy and tenodesis are routine operations for type II superior labrum anterior posterior (SLAP) lesion of the shoulder, but evidence of their superiority is lacking. We conducted this systematic review and meta-analysis to compare the clinical outcomes of arthroscopic repair versus biceps tenotomy and tenodesis intervention.

**Methods:**

The eight studies were acquired from PubMed, Medline, Embase, CNKI, and Cochrane Library. The data were extracted by two of the coauthors independently and were analyzed by RevMan 5.3. Mean differences (MDs), odds ratios (ORs), and 95% confidence intervals (CIs) were calculated. Cochrane Collaboration’s Risk of Bias Tool and Newcastle–Ottawa Scale were used to assess risk of bias.

**Results:**

Eight studies including two randomized controlled trials (RCTs) and six observational studies were assessed. The methodological quality of the trials ranged from low to moderate. The pooled results of UCLA score, SST score, and complications showed that the differences were not statistically significant between the two interventions. The difference of ASES score and satisfaction rate was statistically significant between arthroscopic repair and biceps tenotomy and tenodesis intervention, and arthroscopic biceps tenotomy and tenodesis treatment was more effective. Sensitivity analysis proved the stability of the pooled results, and there were too less included articles to verify the publication bias.

**Conclusions:**

Both arthroscopic repair and biceps tenotomy and tenodesis interventions had benefits in type II SLAP lesions. Arthroscopic biceps tenotomy and tenodesis treatment provides better clinical outcome in ASES score and satisfaction rate and comparable complications compared with arthroscopic repair treatment. In view of the heterogeneity and confounding factors, whether these conclusions are applicable should be further determined in future studies.

## Introduction

The glenoid labrum plays important roles in contributing to stability of the shoulder [1]. The superior glenoid labrum of the shoulder joint, which is related to the intraarticular insertion of the long head of the biceps tendon, is a common site of injury and degeneration [2–4]. When this biceps-labral complex of the glenoid labrum hurts, it caused severe damage to the stability of the shoulder joint, causing instability and pain of the shoulder [5]. Andrews et al. [6] used the term superior labrum anterior posterior (SLAP) to describe these lesions, and Snyder et al. classified the lesions into four subtypes. Among them, type II SLAP lesions occur most frequently [7, 8]. Conservative treatment is not effective, and long-term instability of shoulder can result in articular cartilage injury [9]. The current surgical options for treatment of type II SLAP lesions commonly involve either superior labral repair or biceps tenodesis (and tenotomy). Labral repair is the most common procedure to treat labral tears, but has high rates of complications and poor outcomes [10]. Release of the biceps tendon (tenodesis and tenotomy) is increasingly used as an alternative to SLAP repairs in select patients, but the evidence for it is weak [11]. However, it is still unclear which patients would benefit from either procedure.

Up to now, some clinical studies compared functional outcomes and complications between arthroscopic repair and biceps tenotomy and tenodesis intervention. However, there have been no systematic, quantitative evaluations between the two techniques. In this article, we included eight relevant studies to compare the clinical outcomes of these two techniques in type II SLAP lesions to provide some evidence for clinical decision-making.

## Materials and methods

The work has been reported in line with PRISMA (Preferred Reporting Items for Systematic Reviews and Meta-Analyses) guidelines. Ethical approval and patient consent were not required since the present study was a review of previously published literatures.

### Inclusive criteria of published studies

#### Types of studies

We considered all published and unpublished studies covering randomized controlled trials (RCTs) and observational studies including retrospective and prospective studies.

#### Types of participants

Patients were included in the study if they showed both clinical and radiologic evidence of an isolated type II SLAP lesion with and without a rotator cuff tear and inadequate response to no-operative management (including nonsteroidal anti-inflammatory drugs, physiotherapy, rest, and one local corticosteroid injection), regardless of the gender and age.

Patients with other types of SLAP lesions, such as types I, III, and IV, were excluded from the study. In addition, patients who had an anterior and posterior labral repair were also excluded, as were those who had associated pathology such as biceps tendinopathy and glenohumeral arthritis.

#### Types of interventions

All surgical techniques including the “arthroscopic labral repair and SLAP repair” and “arthroscopic biceps repair and arthroscopic biceps tenotomy and tenodesis technique” were considered. The exclusion criteria were as follows: (1) insufficient clinical outcome data in studies and (2) reviews, letters, and conference articles.

#### Types of outcome measures

The primary outcome measures were the clinical outcomes synthesizing the American Shoulder and Elbow Surgeons (ASES) score, the Shoulder Rating Scale of the University of California at Los Angeles (UCLA) score, the Simple Shoulder Test (SST) score, and the satisfaction rate. The secondary outcomes included complications.

### Search methods for identification of studies

Five databases (PubMed, Medline, Embase, CNKI, and Cochrane Library) were searched using the keywords such as “rotator cuff tear and rotator cuff injuries and rotator cuff tear arthropathy,” “SLAP tear and SLAP lesion and SLAP repair and SLAP rehabilitation and superior labral anterior and posterior lesions,” “biceps tendon and tenodesis and tenotomy,” “surgery and surgical and operation,” and “arthroscopic and arthroscopy” from May 2001 to May 2018 to collect relevant studies about the clinical comparisons of arthroscopic repair versus biceps tenotomy and tenodesis intervention in type II SLAP lesions. The titles and abstracts of potentially related articles identified by the electronic search were reviewed. References from retrieved articles were also assessed to extend the search strategy.

### Data collection and quality assessment

Two partners (TY, WJZ) independently assessed the titles and abstracts of all the studies screened during the initial search, and they excluded any clearly irrelevant studies using the inclusion criteria. Data were independently extracted using a standard data form for the first author’s name, year of publication, sample size, gender, age, intervention, country, study design, follow-up, and relevant outcomes. A third partner (YHD) would handle any disagreement about the inclusion of a study and reach a consensus. Cochrane Collaboration’s Risk of Bias Tool was manipulated for the appraisal of RCT study quality. Observational studies were assessed by the Newcastle–Ottawa Scale including eight items. A higher overall score indicates a lower risk of bias, and a score of 5 and less (out of 9) corresponds to a high risk of bias.

### Statistical analysis

RevMan statistical software 5.3 was used for meta-analysis. The continuous variables would be conducted by mean difference (MD) and 95% confidence interval (CI). For the dichotomous outcome, we calculated the odds ratios (ORs) and 95% CIs. The chi-squared statistic and the *I*^2^ statistic were used for the test of heterogeneity. A *P* < 0.05, *I*^2^ > 50% was considered a significant heterogeneity, and random-effect models were applied. Otherwise, fixed-effect models were used if there was no significant heterogeneity (*P* ≥ 0.05, *I*^2^ ≤ 50%). We also performed sensitivity analysis by omitting one study at a time to test the stability of the pooled results. Publication bias was showed by the funnel plot.

## Results

### Study identification and inclusion

**Fig. 1:**
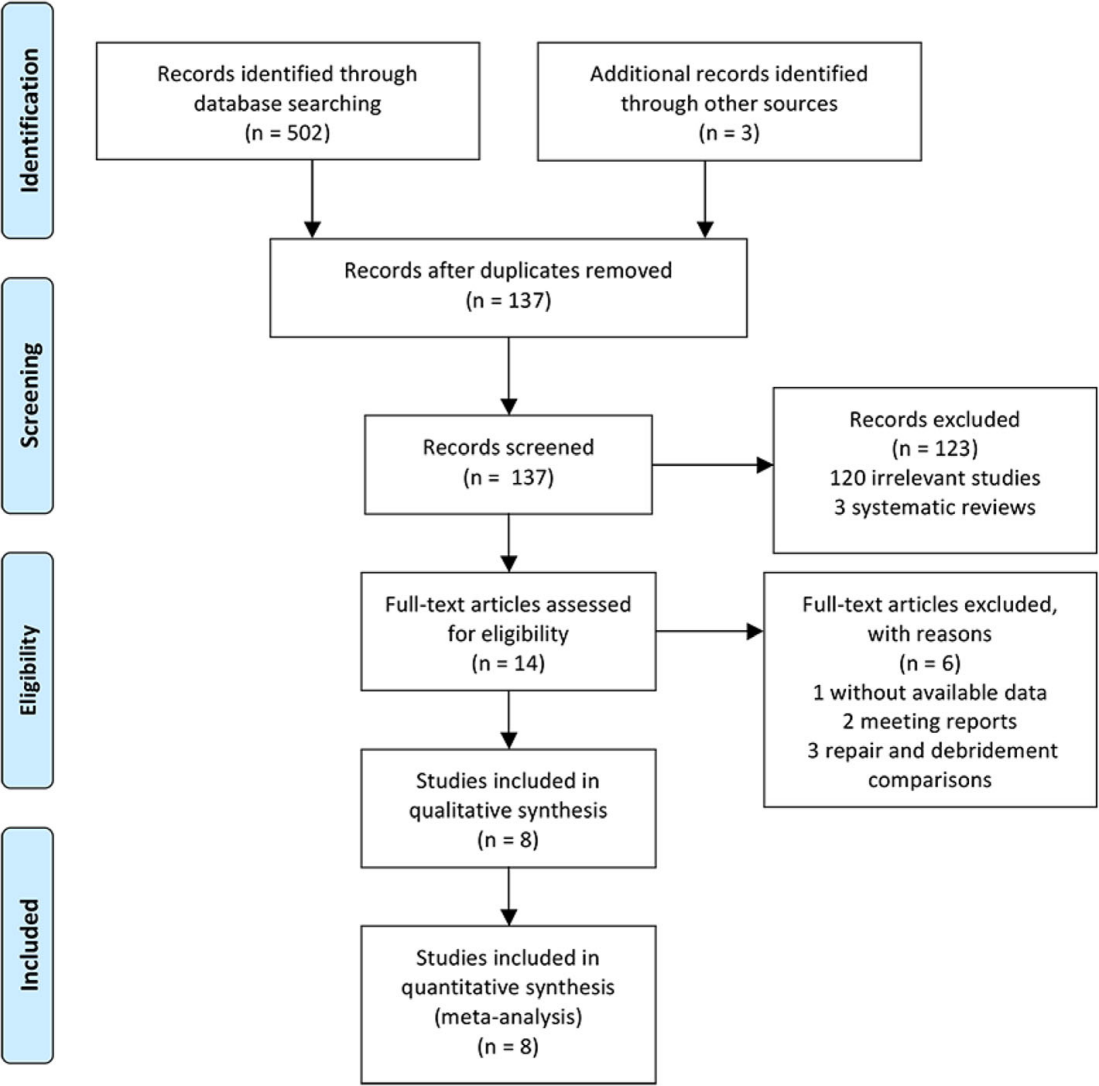
PRISMA flow diagram

Searches conducted in the PubMed, Medline, Embase, CNKI, and Cochrane Library databases and other sources yielded a total of 505 articles. After removing duplicates, 137 literatures were remained. Based on the title and abstract review, 120 irrelevant articles and 3 systematic reviews of them were excluded. Fourteen full-text articles were assessed for eligibility. However, six articles were excluded based on the previously established exclusion criteria (one without available data, two meeting reports, and three repair and debridement comparisons). Finally, eight trials (two RCTs and six observational studies) were included in this systematic review and meta-analysis. The detail of selection process is listed in Fig. 1.

### Study characteristics

We assessed eight studies [12–19] including two RCTs and six retrospective studies in this article. The included studies were conducted in seven countries (Norway, China, the USA, Australia, Korea, France, Italy) from 2008 to 2017 and involved 325 patients (162 patients treated with arthroscopic repair technique, 163 patients treated with arthroscopic biceps tenotomy and tenodesis technique) aged 31 to 64.7 years. The average follow-up duration ranged from 12 to 41.1 months. The clinical outcomes of the studies were evaluated mainly based on ASES score, UCLA score, SST score, satisfaction rate, and complications. The detailed information of included studies is shown in Table 1.

**Table 1:** Characteristics of studies included

	Year	Sample size (R/BT)	Female (%)	Mean age (years)	Intervention		Country	Study design	Follow-up (month)	Relevant outcome
					R	BT				
Schrøder et al. [12]	2017	40/39	R 37.5%	R 40 (22–57)	Labral repair	Biceps tenodesis	Norway	RCT	24	Rowe; WOSI; OISS; EQ-5D; satisfaction rate; EQ-VAS
			BT 38.5%	BT 40 (18–64)						
Chen et al. [13]	2016	11/11	R 27.3%	R 40.36 ± 3.98	Labral repair	Biceps tenotomy and tenodesis	China	Retrospective study	12	UCLA; ASES
			BT 36.4%	BT 41.91 ± 3.11						
Zhao et al. [14]	2015	22/16	R 36.4%	R 49 ± 2.8	SLAP repair	Biceps tenodesis	China	Retrospective case-control study	24	UCLA; SST
			BT 43.8%	BT 49.3 ± 3.7						
Denard et al. [15]	2014	22/15	R 27.3%	R 45.2 ± 5.5	Biceps repair	Biceps tenodesis	USA	Retrospective study	R 63.2 ± 14.5	Satisfaction rate; complications
			BT 13.3%	BT 52.0 ± 8.0					BT 41.1 ± 19.8	
Ek et al. [16]	2014	10/15	R 0%	R 31(21–43)	SLAP repair	Biceps tenodesis	Australia	Retrospective study	R 35 (25–52)	SSV; VAS; ASES; satisfaction rate; complications
			BT 6.7%	BT 47(30–59)					BT 31 (26–43)	
Kim et al. [17]	2012	16/20	R 56.3%	R 61.1 ± 5.1	SLAP repair	Biceps tenotomy	Korea	Retrospective study	24	SST; ASES; UCLA
			BT 55%	BT 63.3 ± 6.0						
Boileau et al. [18]	2009	10/15	R 0%	R 37(19–57)	SLAP repair	Biceps tenodesis	France	Retrospective study	R 35 (24–69)	Satisfaction rate; complications; reoperation
			BT 40%	BT 52(28–64)					BT 34 (24–68)	
Franceschi et al. [19]	2008	31/32	R 41.9%	R 61.8 (51–79)	SLAP repair	Biceps tenotomy	Italy	RCT	34.8	UCLA; ROM; operation time; complications
			BT 53.1%	BT 64.7 (53–81)						

*R* repair; *BT* biceps tenotomy and tenodesis; *UR* un-reported; *RCT* randomized controlled trial; *VAS* visual analogue scale; *UCLA* the University of California, Los Angeles Score; *ASES* the American Shoulder and Elbow Surgeons; *SST* the Simple Shoulder Test; *SSV* the subjective shoulder value; *WOSI* the Western Ontario Shoulder Instability Index; *OISS* the Oxford Instability Shoulder Score; *EQ* EuroQol; *ROM* range of motion

### Methodological assessment of study quality

**Fig. 2:**
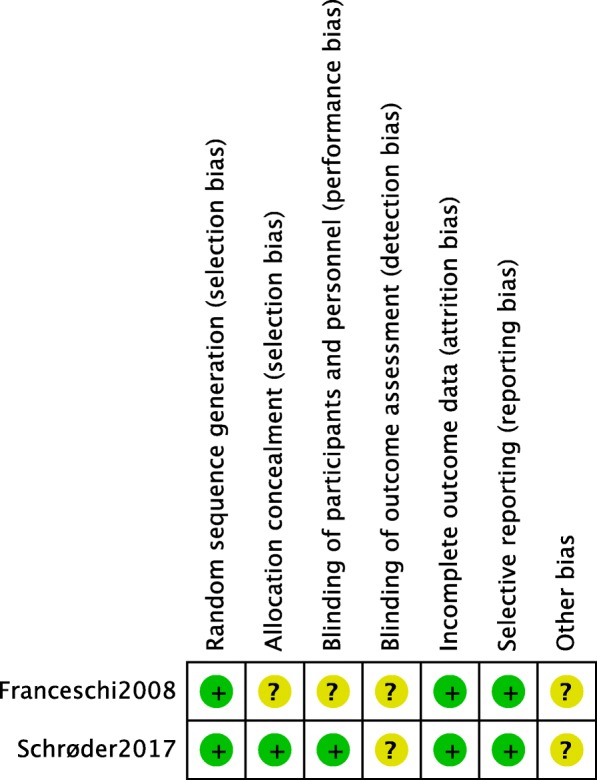
Risk of bias summary: this risk of bias tool incorporates the assessment of randomization (sequence generation and allocation concealment), blinding (participants and outcome assessors), incomplete outcome data, selective outcome reporting, and other risk of bias. The items were judged as “low risk,” “unclear risk,” and “high risk.” Green means “low risk,” red means “high risk,” and yellow means “unclear risk”

Methodological quality assessment of the seven included studies is presented in Fig. 2 and Table 2. Among the RCTs, Schrøder et al.’s study [12] clearly described the random sequence generation by the permuted block method, and the blinding and allocation concealment were mentioned, which could be regarded as a high-quality study. However, Franceschi et al. [18] did not describe any blinding and allocation concealment, which could be regarded as a low-quality study. Among the observational studies, the Newcastle–Ottawa Scale including the exposed cohort, the non-exposed cohort, ascertainment of exposure, outcome of interest, comparability, assessment of outcome, length of follow-up, and adequacy of follow-up was used to assess the risk of bias. The scores of all six studies ranged from 7 to 8, indicating a low risk of bias.

**Table 2:** Risk of bias was assessed using the Newcastle–Ottawa Scale

Study	Selection			Outcome of interest	Comparability	Selection			Total score
	Exposed cohort	Non-exposed cohort	Ascertainment of exposure			Assessment of outcome	Length of follow-up	Adequacy of follow-up	
Chen et al. [13]	*	*	*	*	*	*	*	–	7
Zhao et al. [14]	*	*	*	*	*	*	*	*	8
Denard et al. [15]	*	*	*	*	*	*	*	*	8
Ek et al. [16]	*	*	*	*	*	*	*	*	8
Kim et al. [17]	*	*	*	*	*	*	*	*	8
Boileau et al. [18]	*	*	*	*	*	*	*	*	8

*Risk of bias was assessed using the Newcastle–Ottawa Scale. A higher overall score indicates a lower risk of bias; a score of 5 or less (out of 9) corresponds to a high risk of bias

### Comparison of ASES score between arthroscopic repair and biceps tenotomy and tenodesis

**Fig. 3:**
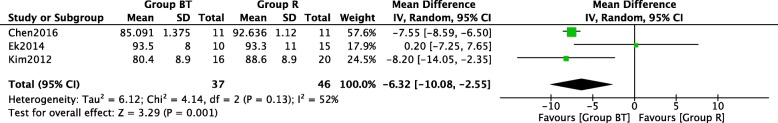
Forest plot of comparison: ASES score between arthroscopic repair (group R) and biceps tenotomy and tenodesis (group BT) technique

Comparison of postoperative ASES score between arthroscopic repair and biceps tenotomy and tenodesis was conducted between the three included studies [13, 16, 17], which enrolled 83 patients (37 patients receiving arthroscopic repair and 46 patients receiving arthroscopic biceps tenotomy and tenodesis), as shown in Fig. 3. Heterogeneity testing showed that there was moderate heterogeneity between the studies (*P* = 0.13, *I*^2^ = 52%), so the random-effect model was used to pool the data for the two groups. The overall estimate showed that the difference was statistically significant between the two groups (MD = − 6.32, 95% CI = − 10.08 to − 2.55, *P* = 0.001).

### Comparison of UCLA score between arthroscopic repair and biceps tenotomy and tenodesis

**Fig. 4:**
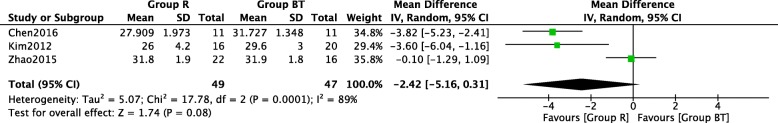
Forest plot of comparison: UCLA score between arthroscopic repair (group R) and biceps tenotomy and tenodesis (group BT) technique

In Fig. 4, three included studies [13, 14, 17] consisting of 96 patients (49 patients received arthroscopic repair treatment and 47 patients received arthroscopic biceps tenotomy and tenodesis treatment) investigated postoperative UCLA score. High heterogeneity among studies (*P* = 0.0001, *I*^2^ = 89%) was found, so we used the random-effect model to pool the data. The overall estimate indicated that the pooled MD was − 2.42 (95% CI = − 5.16–0.31, *P* = 0.08), suggesting that these two treatments had no statistically significant difference.

### Comparison of SST score between arthroscopic repair and biceps tenotomy and tenodesis

**Fig. 5:**
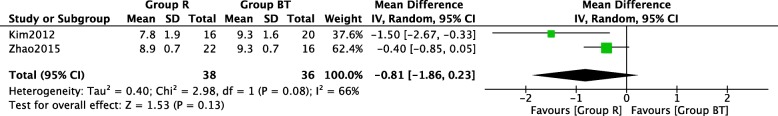
Forest plot of comparison: SST score between arthroscopic repair (group R) and biceps tenotomy and tenodesis (group BT) technique

Comparison of postoperative SST score between arthroscopic repair and biceps tenotomy and tenodesis was conducted among the two included studies [14, 17], which included 74 patients (38 patients receiving arthroscopic repair and 36 patients receiving arthroscopic biceps tenotomy and tenodesis), as shown in Fig. 5. Heterogeneity testing showed that there was moderate heterogeneity among the studies (*P* = 0.08, *I*^2^ = 66%), so the random-effect model was used to pool the data from the two studies. The pooled result showed that the difference was not statistically significant between the two groups (MD = − 0.81, 95% CI = − 1.86–0.23, *P* = 0.13).

### Comparison of satisfaction rate between arthroscopic repair and biceps tenotomy and tenodesis

**Fig. 6:**
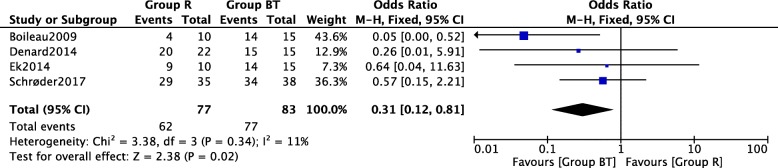
Forest plot of comparison: satisfaction rate between arthroscopic repair (group R) and biceps tenotomy and tenodesis (group BT) technique

Four included studies [12, 15, 16, 19] including 77 arthroscopic repair surgery group cases and 83 arthroscopic biceps tenotomy and tenodesis surgery group cases provided the data in terms of postoperative satisfaction rate. A heterogeneity test revealed that low significant heterogeneity existed among the studies (*P* = 0.34, *I*^2^ = 11%) and the fixed-effect model was used. A pooled analysis revealed that there was significant difference between these two surgery groups (OR = 0.31, 95% CI = 0.12–0.81, *P* = 0.02) as shown in Fig. 6.

### Comparison of complications between arthroscopic repair and biceps tenotomy and tenodesis

**Fig. 7:**
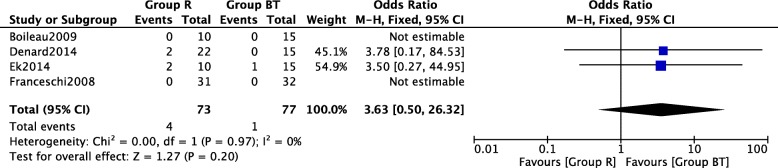
Forest plot of comparison: complications between arthroscopic repair (group R) and biceps tenotomy and tenodesis (group BT) technique

In Fig. 7, four included studies [15, 16, 18, 19] consisting of 150 SLAP lesion patients (73 patients received arthroscopic repair and 77 patients received arthroscopic biceps tenotomy and tenodesis technique) reported complications. No heterogeneity among studies (*P* = 0.97, *I*^2^ = 0%) was found, so we used the fixed-effect model. The overall estimate indicated that the pooled OR was 3.63 (95% CI = 0.50–26.32, *P* = 0.20), suggesting that the difference was not statistically significant.

### Sensitivity analysis and publication bias

**Fig. 8:**
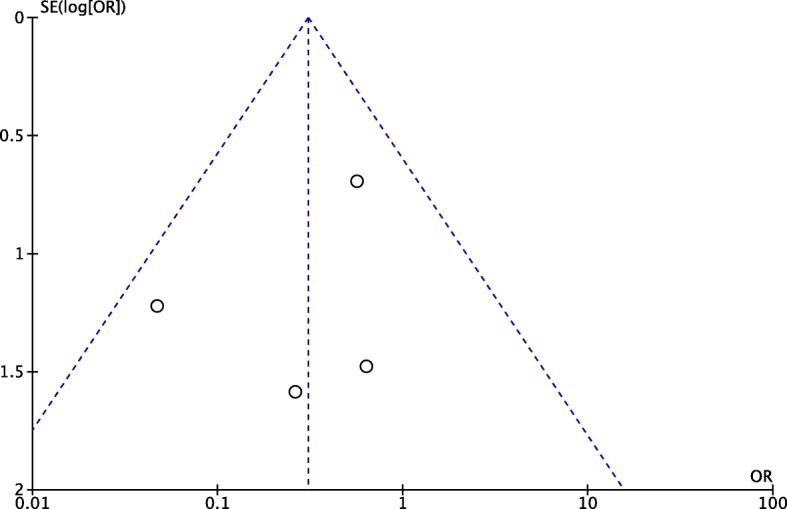
Funnel plot to test for publication bias. Each point represents a separate study for the indicated association. The vertical line represents the mean effects size. OR odds ratio, SE standard error

We performed a sensitivity analysis to assess the stability of the pooled results. Among the most studies, the heterogeneity results were not obviously altered after sequentially omitting each study, indicating that our results were statistically reliable. The funnel plot of the included studies is shown in Fig. 8. The points in the funnel plot were almost symmetrically distributed. However, too less included articles lead to an unbelievable result, and the publication bias could not be ignored.

## Discussion

### Summary of main results

In this study, we identified two RCTs and six observational studies for investigating the clinical outcomes of arthroscopic repair versus biceps tenotomy and tenodesis intervention. Our meta-analysis results showed that the differences were not statistically significant between the two interventions for UCLA score, SST score, and complications. However, a different result was discovered by ASES score and satisfaction rate analysis. The difference of the ASES score and satisfaction rate was statistically significant between the two interventions, and the arthroscopic biceps tenotomy and tenodesis technique proved it had a higher efficacy. Long-term evaluation is still necessary.

The importance of the superior labral complex (including the long head of the biceps) in overall glenohumeral stabilization and shoulder function has been debated and continues to be controversial [20, 21]. Patzer et al. in a biomechanical study showed the stabilizing effect of the superior labral complex is dependent on the attached long head of the biceps tendon, with bicep tenotomy and SLAP repair resulting in increased glenohumeral translation [22]. However, arthroscopic biceps tenodesis does not result in proximal humeral migration and anterior instability. More importantly, by removing a pain generator, it may be possible to restore normal kinematics to the athlete’s shoulder [23]. Another debate is whether arthroscopic biceps tenotomy should be with and without tenodesis. The disadvantages of biceps tenotomy may be distal migration of the long head of the biceps tendon with cosmetic deformity (Popeye sign) and significantly impaired shoulder strength. However, it is a quick procedure that does not require fixation [24]. What is more, Osbahr et al. reported on the cosmetic appearance of tenotomy versus tenodesis. The results revealed that there was no significant difference in the patients’ self-rated levels of anterior shoulder pain, cosmetic deformity, and muscle spasm between the two groups [25]. In addition, to date, there is still no clear consensus on the patient age for SLAP lesions. Some authors advocate that SLAP repair should be reserved for the young and active patient [26]. It has been the senior doctor’s preference to perform arthroscopic SLAP repairs for patients who were generally younger (< 35 years) and/or those in whom healthy labral tissue was found at the time of arthroscopy. In contrast, for patients who were generally older (> 35 years) and/and those with degenerative and frayed labrums, biceps tenodesis was preferred. However, in Schrøder et al.’s study of patients who had superior labral repairs for isolated type II lesions with long-term follow-up, no difference was observed between older patients (> 40 years) and younger patients (< 40 years) in terms of overall satisfaction and functional outcome scores [27]. No similar comparison study is conducted for biceps tenotomy and tenodesis between older patients and younger patients.

The complications in eight included studies also should be discussed. On the whole, four (5.5%) complications under arthroscopic repair were reported and one (1.3%) complication under arthroscopic biceps tenotomy and tenodesis was reported in four included studies [15, 16, 18, 19]. Ek et al. reported that in the tenodesis group, there was one failure of the tenodesis, which presented as a clear “Popeye” deformity; in the SLAP repair group, postoperative stiffness occurred in two cases, who were treated conservatively with physical therapy and subsequently resolved [16]. Denard et al. showed two patients in the repair group required a subsequent capsular release for persistent postoperative stiffness [15]. No intraoperative complications, nerve deficits, and wound infections occurred in any patient.

### Limitations of the study

Some limitations of this study should be noted. First, the small sample size and age matching might have affected the significant difference between the two surgical procedures. Second, significant statistical heterogeneity of ASES score, UCLA score, and SST score still existed among the included trials, which may be explained by the clinical diversity among trials. Third, our study only included four articles for conducting funnel plot and the publication bias could not be ignored. Last but not least, the included studies were mostly observational studies and not RCTs, and they largely relied on retrospectively collected data, resulting in a high risk of selection bias. More large-sample, multi-center, high-quality, randomized controlled trials are needed to verify the outcomes of this meta-analysis.

## Conclusions

In conclusion, both arthroscopic repair and biceps tenotomy and tenodesis interventions had benefits in type II SLAP lesions. Arthroscopic biceps tenotomy and tenodesis treatment provides better clinical outcome in ASES score and satisfaction rate and comparable complications compared with arthroscopic repair treatment. In view of the heterogeneity and confounding factors, whether these conclusions are applicable should be further determined in future studies.

## Abbreviations

ASES: American Shoulder and Elbow Surgeons
CI: Confidence interval
MD: Mean difference
ORs: Odds ratios
PRISMA: Preferred Reporting Items for Systematic Reviews and Meta-Analyses
RCTs: Randomized controlled trials
SLAP: Superior labrum anterior posterior
SST: Simple Shoulder Test
UCLA: University of California at Los Angeles
